# Challenges in AI-driven Biomedical Multimodal Data Fusion and Analysis

**DOI:** 10.1093/gpbjnl/qzaf011

**Published:** 2025-02-27

**Authors:** Junwei Liu, Xiaoping Cen, Chenxin Yi, Feng-ao Wang, Junxiang Ding, Jinyu Cheng, Qinhua Wu, Baowen Gai, Yiwen Zhou, Ruikun He, Feng Gao, Yixue Li

**Affiliations:** Guangzhou National Laboratory, Guangzhou 510005, China; Guangzhou National Laboratory, Guangzhou 510005, China; College of Life Sciences, University of Chinese Academy of Sciences, Beijing 100049, China; HIM-BGI Omics Center, Hangzhou Institute of Medicine, Chinese Academy of Sciences, Hangzhou 310022, China; Guangzhou National Laboratory, Guangzhou 510005, China; School of Intelligent Systems Engineering, Sun Yat-sen University, Shenzhen 518107, China; Guangzhou National Laboratory, Guangzhou 510005, China; Key Laboratory of Systems Health Science of Zhejiang Province, School of Life Science, Hangzhou Institute for Advanced Study, University of Chinese Academy of Sciences, Hangzhou 310024, China; Guangzhou National Laboratory, Guangzhou 510005, China; Key Laboratory of Systems Health Science of Zhejiang Province, School of Life Science, Hangzhou Institute for Advanced Study, University of Chinese Academy of Sciences, Hangzhou 310024, China; Guangzhou National Laboratory, Guangzhou 510005, China; Multimedia Laboratory, The Chinese University of Hong Kong, Hong Kong Special Administrative Region 999077, China; Guangzhou National Laboratory, Guangzhou 510005, China; Key Laboratory of Systems Health Science of Zhejiang Province, School of Life Science, Hangzhou Institute for Advanced Study, University of Chinese Academy of Sciences, Hangzhou 310024, China; Department of Colorectal Surgery, The Sixth Affiliated Hospital, Sun Yat-sen University, Guangzhou 510655, China; Biomedical Innovation Center, The Sixth Affiliated Hospital, Sun Yat-sen University, Guangzhou 510655, China; School of Electronics and Communication Engineering, Sun Yat-sen University, Shenzhen 518107, China; BYHEALTH Institute of Nutrition & Health, Guangzhou 510663, China; Department of Colorectal Surgery, The Sixth Affiliated Hospital, Sun Yat-sen University, Guangzhou 510655, China; Biomedical Innovation Center, The Sixth Affiliated Hospital, Sun Yat-sen University, Guangzhou 510655, China; Shanghai Artificial Intelligence Laboratory, Shanghai 200433, China; Guangzhou National Laboratory, Guangzhou 510005, China; Key Laboratory of Systems Health Science of Zhejiang Province, School of Life Science, Hangzhou Institute for Advanced Study, University of Chinese Academy of Sciences, Hangzhou 310024, China; GZMU-GIBH Joint School of Life Sciences, The Guangdong-Hong Kong-Macau Joint Laboratory for Cell Fate Regulation and Diseases, Guangzhou Medical University, Guangzhou 511436, China; School of Life Sciences and Biotechnology, Shanghai Jiao Tong University, Shanghai 200240, China; Shanghai Institute of Nutrition and Health, Chinese Academy of Sciences, Shanghai 200030, China; Collaborative Innovation Center for Genetics and Development, Fudan University, Shanghai 200433, China; Shanghai Institute for Biomedical and Pharmaceutical Technologies, Shanghai 200032, China

**Keywords:** Multimodal learning, Biomedical analysis, Large language model, Model interpretation, Meta-learning

## Abstract

The rapid development of biological and medical examination methods has vastly expanded personal biomedical information, including molecular, cellular, image, and electronic health record datasets. Integrating this wealth of information enables precise disease diagnosis, biomarker identification, and treatment design in clinical settings. Artificial intelligence (AI) techniques, particularly deep learning models, have been extensively employed in biomedical applications, demonstrating increased precision, efficiency, and generalization. The success of the large language and vision models further significantly extends their biomedical applications. However, challenges remain in learning these multimodal biomedical datasets, such as data privacy, fusion, and model interpretation. In this review, we provide a comprehensive overview of various biomedical data modalities, multimodal representation learning methods, and the applications of AI in biomedical data integrative analysis. Additionally, we discuss the challenges in applying these deep learning methods and how to better integrate them into biomedical scenarios. We then propose future directions for adapting deep learning methods with model pretraining and knowledge integration to advance biomedical research and benefit their clinical applications.

## Graphical Abstract

**Figure:**
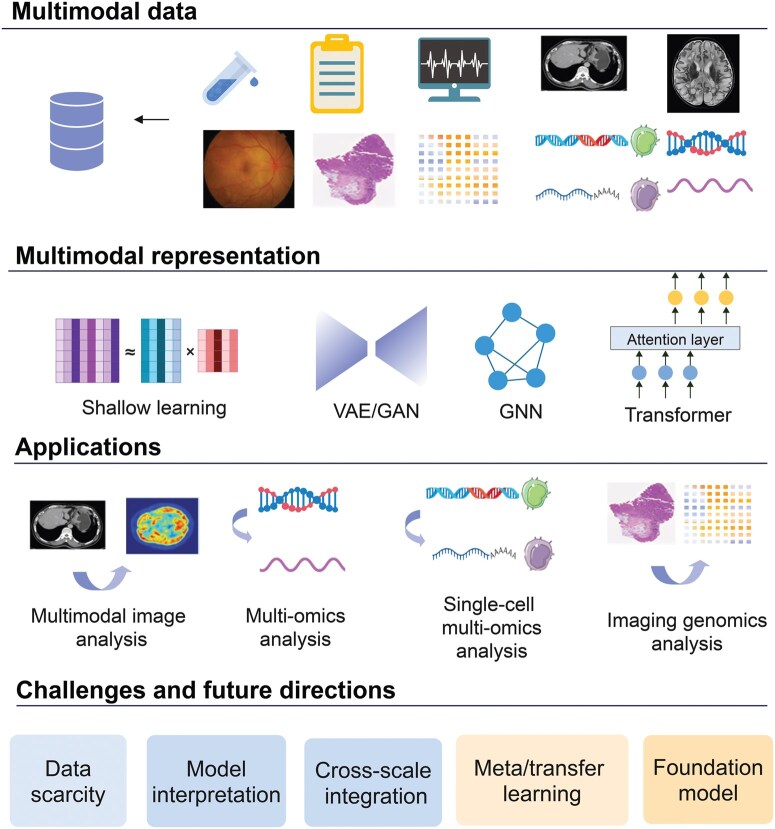

## Introduction

The development of biological and medical examination methods has significantly expanded the scope of personal biomedical information, which ranges from genomics, transcriptomics, proteomics, and metabolomics to radiology and electronic health records (EHRs) [1]. Single or unified multimodal datasets have been utilized in clinical usage for disease diagnosis, individual treatment, risk stratification, and so on. Moreover, the advent of single-cell profiling methods, including single-cell RNA sequencing (scRNA-seq), single-cell assay for transposase-accessible chromatin with sequencing (scATAC-seq), cellular indexing of transcriptomes and epitopes by sequencing (CITE-seq), and spatial transcriptomics, has enhanced our understanding of various biological processes in human development and tumorigenesis at the cellular level [2]. In addition to the comprehensive insights of both clinical and molecular measures into patients, how to integrate their information for precise disease diagnosis, new biomarker identification, treatment, and drug design presents a crucial challenge in the field.

Artificial intelligence (AI) techniques have been intensively integrated into different biomedical applications, such as medical image analysis, disease diagnosis, public health, protein design, and others [3]. In medical image analysis, deep learning methods have been broadly utilized to extract complementary tissue structure or morphological features of images to aid in lesion detection, segmentation, and computer-assisted diagnosis [4]. These image diagnostic methods have been demonstrated to be more productive and accurate, which aids in fast decision-making in clinical settings [5]. In biology applications, deep learning has been employed to learn the structure of DNA and protein sequences [6,7], predict protein structure [8], simulate and predict the genomic mutation risks [9], and facilitate drug discovery [10]. In single-cell analysis, the high-throughput single-cell measure techniques have generated millions of individual cell data points, which are well-suited for applying deep learning methods to multiple tasks, such as atlas-level data integration [11], cellular annotation [12], and gene expression learning in single cells [13–15]. Furthermore, the success of large language models (LLMs) has opened up new opportunities for integrating medical domain knowledge to develop foundation models for automatically generating radiology reports, suggesting medical intervention, providing medical advice to patients, and being capable of handling more other new tasks in the biomedical field [16].

The growth of various diagnostic methods has contributed to the generation of multiple datasets encompassing individual patients, tissues, and cells. Despite individual datasets capturing distinct phenotypic changes and associated factors, the validation of causal regulatory mechanisms and the pursuit of precise interventions in the most suitable targets still necessitate the incorporation of additional modalities and the need for computational methods for multimodal data integration. In oncology applications, the radiological images and genomic information of cancer patients have been integrated for enhanced prognosis prediction and patient classification [17]. The integration of multi-omics datasets with the drug usage information has been used to identify drug-associated individual omics features, quantifying the drug response effects [18]. In digital pathology applications, whole slide images have been used for predicting genomic features [19] and integrated with genomic features for prognosis prediction [20]. In single-cell multi-omics applications, these multiple profiles have been used to characterize the cellular and spatiotemporal genomic regulations [21]. Various methods have been developed to integrate single cells with different omics [22], uncover the regulatory networks of single-cell datasets [23], or combine gene expression profiles with spatial information [24]. Collectively, the development of both multimodal data collection methods and data integration algorithms significantly enhances the delineation of various biomedical progresses and offers more robust feature attribution analysis.

Despite the advancement of multimodal learning in the biomedical field, various challenges can hinder model training and its further applications [1]. Data challenge is universally prevalent in model training of biomedical data. Due to privacy restrictions, sharing datasets across individual institutes is infeasible, which further limits the scale of training datasets [25]. Meanwhile, different data acquisition methods and incomplete multimodal datasets further require complex data preprocessing and tailored model architectures that can handle incomplete model training [26]. Besides, data interpretation is crucial in deep learning analysis of multimodal biomedical datasets [27]. The understanding of important gene features or cross-modality regulatory networks is essential to uncover the mechanism of disease development and identify new disease biomarkers and drug targets [28]. Furthermore, how to integrate additional biomedical knowledge into multimodal deep learning models and apply *in silico* perturbation prediction for cross-modality regulation validation still requires further discussion [29,30].

Several reviews have discussed the methods, applications, and challenges for multimodal biomedical data fusion. Acosta et al. highlighted the application of multimodal biomedical AI in health surveillance and personalized medicine [1], but did not comprehensively review fusion methods. Stahlschmidt et al. summarized multimodal fusion methods in biomedical analysis [31], and Duan et al. provided detailed review of multimodal learning methods for different biomedical data modalities potentially applicable in clinical scenarios [32]. However, existing reviews are limited by the scale of multimodal biomedical data and lack discussions on their role in advancing biological insights into human diseases. Additionally, the recent rise of LLMs underscores the need to rethink and redefine the future of biomedical multimodal analysis.

In this review, we extend the discussion considering the scale of multimodal biomedical data, summarize the existing multimodal biomedical data learning methods, and explore the prospects and challenges when integrating these methods into specific biomedical scenarios. Firstly, we summarize existing multimodal biomedical data by category and list available data resources. Then, several data representation learning methods and their roles in multimodal learning are reviewed. Next, we explore the applications of deep learning methods in several important aspects of multimodal biomedical analysis, including clinical multimodal data integration, multi-omics analysis, single-cell analysis, and genotype–phenotype association analysis. After that, we characterize the challenges in multimodal learning for biomedical datasets, such as data privacy, model interpretation, and cross-scale data integration. Lastly, we propose the future directions for biomedical multimodal learning, including the use of meta and transfer learning for limited cohort datasets in clinical settings, the adaption of LLMs to integrate biomedical knowledge, and the implementation of automated knowledge queries for improved representation learning in multimodal datasets, all aiming at advancing biomedical research.

## Multimodal biomedical data

**Figure 1:**
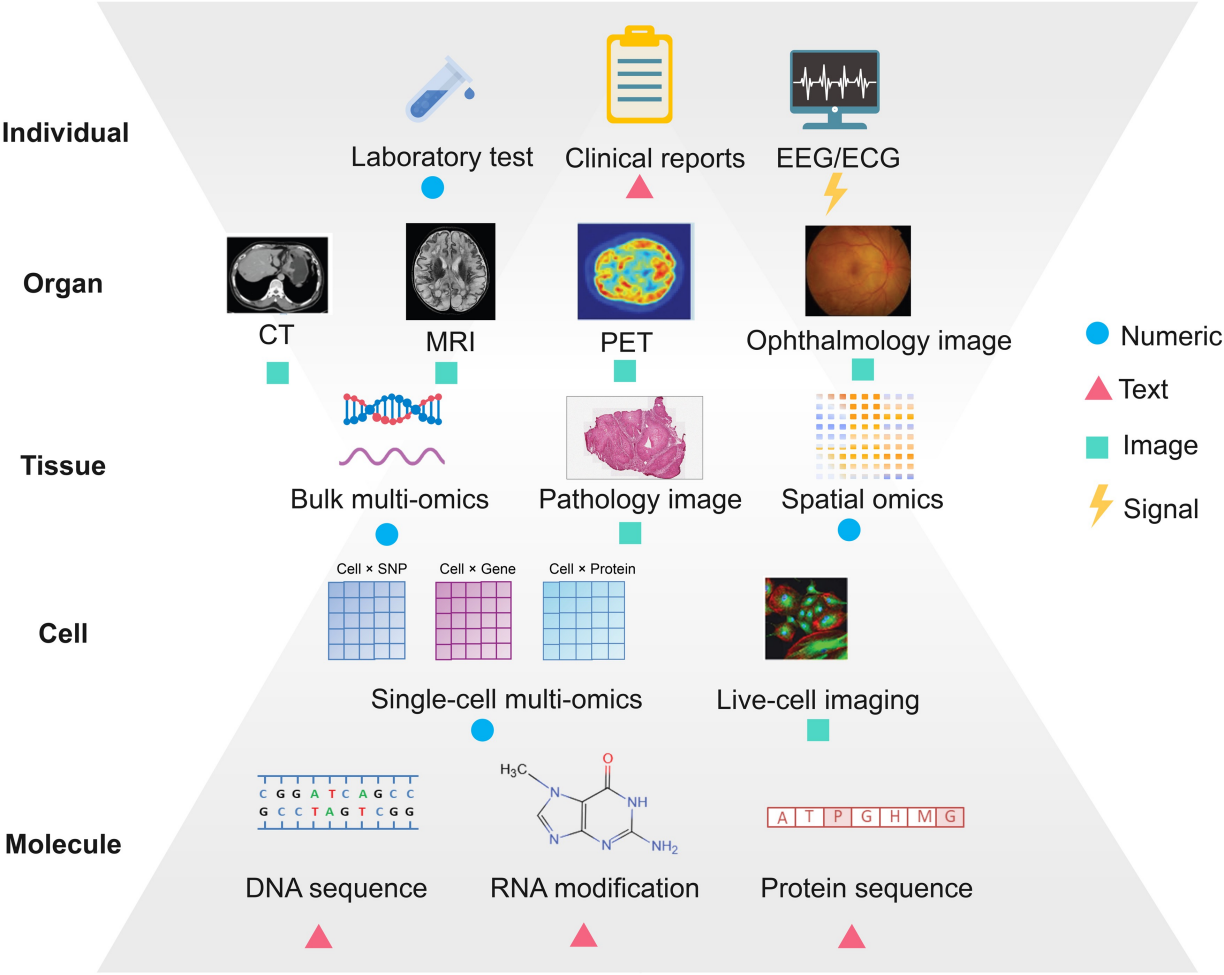
The categories and scales of biomedical multimodal data Biomedical multimodal datasets vary across individual, organ, tissue, cell, and molecule levels from biology perspective (see the annotations on the left). From computational terms, these datasets are categorized into numeric, image, text, and sequence data types (see the annotations on the right). EEG, electroencephalogram; ECG, electrocardiogram; CT, computed tomography; MRI, magnetic resonance imaging; PET, positron emission tomography; SNP, single nucleotide polymorphism.

Multimodal biomedical datasets have been rapidly accumulating, offering extensive resources for in-depth biomedical research (**Table 1**). These datasets vary widely across scales and types (**Figure 1**), encompassing numeric data, image data, text data, and sequence data. Additionally, based on their sources, these modalities can be grouped into sequencing data, clinical data, and experimental data. The various data modalities are summarized below by category, along with a discussion of the relevant processing methods for each.

**Table 1:** Representative multimodal biomedical databases

Database	Modality	Website	Category
TCGA, TCIA	Multi-omics, biomedical image	https://www.cancer.gov/ccg/research/genome-sequencing/tcga;https://www.cancerimagingarchive.net/	Mixed data
CPTAC	Proteogenomics, histopathology image	https://proteomics.cancer.gov/programs/cptac	Mixed data
ICGC	Multi-omics	https://docs.icgc-argo.org/docs/data-access/icgc-25k-data	Sequencing data
OrganoidBase	Multi-omics	https://organoid.crownbio.com/OrganoidModels/Index	Sequencing data
SODB	Spatial multi-omics	https://gene.ai.tencent.com/SpatialOmics	Sequencing data
Single Cell Atlas	Single-cell multi-omics	https://www.singlecellatlas.org/	Sequencing data
ADNI	Multimodal image	https://adni.loni.usc.edu/	Clinical data
OASIS	Clinical information, MRI image, omics	https://sites.wustl.edu/oasisbrains/	Mixed data
UK Biobank	Image, genomics, EHR	https://www.ukbiobank.ac.uk/	Mixed data
CNCB	Multi-omics, image, clinical information	https://www.cncb.ac.cn/	Mixed data
TOPMed	Multi-omics, image, clinical information	https://topmed.nhlbi.nih.gov/	Mixed data
GECIP	Genomics, clinical information, image	https://research.genomicsengland.co.uk/	Mixed data
/	Multimodal EEG data	https://reshare.ukdataservice.ac.uk/854301/	Experimental data
MedMNIST	Multimodal medical image	https://doi.org/10.5281/zenodo.10519652	Clinical data
BraTS	Multimodal MRI image	https://www.synapse.org/Synapse:syn51156910	Clinical data
RP3D-Diag	Multimodal medical image	https://huggingface.co/datasets/QiaoyuZheng/RP3D-DiagDS	Clinical data
IDR	Multimodal image	https://idr.openmicroscopy.org/	Experimental data
BBBC	Microscopy image	https://bbbc.broadinstitute.org/	Experimental data

*Note*: MRI, magnetic resonance imaging; EHR, electronic health record; EEG, electroencephalogram.

### Numeric data

Multi-omics data generated by sequencing typically take the form of numeric matrices, representing features like genome mutation states, transcriptome expression, and protein expression at the tissue or cell level. However, these sequencing data are often noisy and sparse, posing challenges for analysis. To address these issues, various methods have been developed. For instance, in scRNA-seq analysis, zero-inflated autoencoders were introduced to handle data sparsity by employing a specialized loss function that aligns with the binomial distribution of single-cell data [33]. In clinical settings, patients frequently undergo laboratory tests, such as blood tests and genomic assessments, which generate additional numeric data. Other baseline information, including age and gender, is also relevant for clinical decision-making. For multimodal analysis, these clinical data are often incorporated directly into networks without requiring additional preprocessing.

### Image data

Imaging is a fundamental tool in clinical examinations, often utilized throughout the entire patient management process. Non-invasive imaging techniques such as computed tomography (CT), magnetic resonance imaging (MRI), and ultrasound are widely used for disease diagnosis. For specific organs, additional imaging methods are employed for screening or diagnosis, including endoscopy for gastrointestinal conditions [34] and fundus imaging for eye diseases [35]. Moreover, in most clinical settings, histopathology imaging is considered the “gold standard”, as it provides high-resolution views of tissue, including detailed cellular morphology [36]. Beyond clinical imaging, live-cell imaging plays a crucial role in biological experiments, offering insights into cellular activities [37]. These imaging data are complex to process, necessitating specialized algorithms for tasks such as image denoising, cell segmentation, and super-resolution enhancement.

### Text data

Clinical reports are comprehensive documents that capture patient histories, diagnostic findings, treatment plans, and progress notes. Due to their unstructured or semi-structured nature, analyzing these reports systematically poses a challenge. To address this, natural language processing (NLP) techniques such as named entity recognition (NER), text classification, and relation extraction are used [38]. These methods facilitate the extraction of valuable insights, supporting improved patient care and advancing research. Similarly, molecular data, including DNA, RNA, and protein sequences, are unstructured and exhibit their forms of grammar and semantics. NLP techniques, adapted to capture these biological patterns, are also applied to these sequences. Methods like *k*-mer splitting [39], among others [40], have been developed to better interpret the biological grammar and semantics inherent in molecular data.

### Signal data

Biomedical signals such as electrocardiogram (ECG) and electroencephalogram (EEG) are forms of time-series data, distinct from other data types and requiring specialized processing approaches [41]. Traditional methods often use signal decomposition to analyze these signals based on their frequency components. Additionally, these time-series data can be processed similarly to image data in certain applications.

## Multimodal representation learning methods

**Figure 2:**
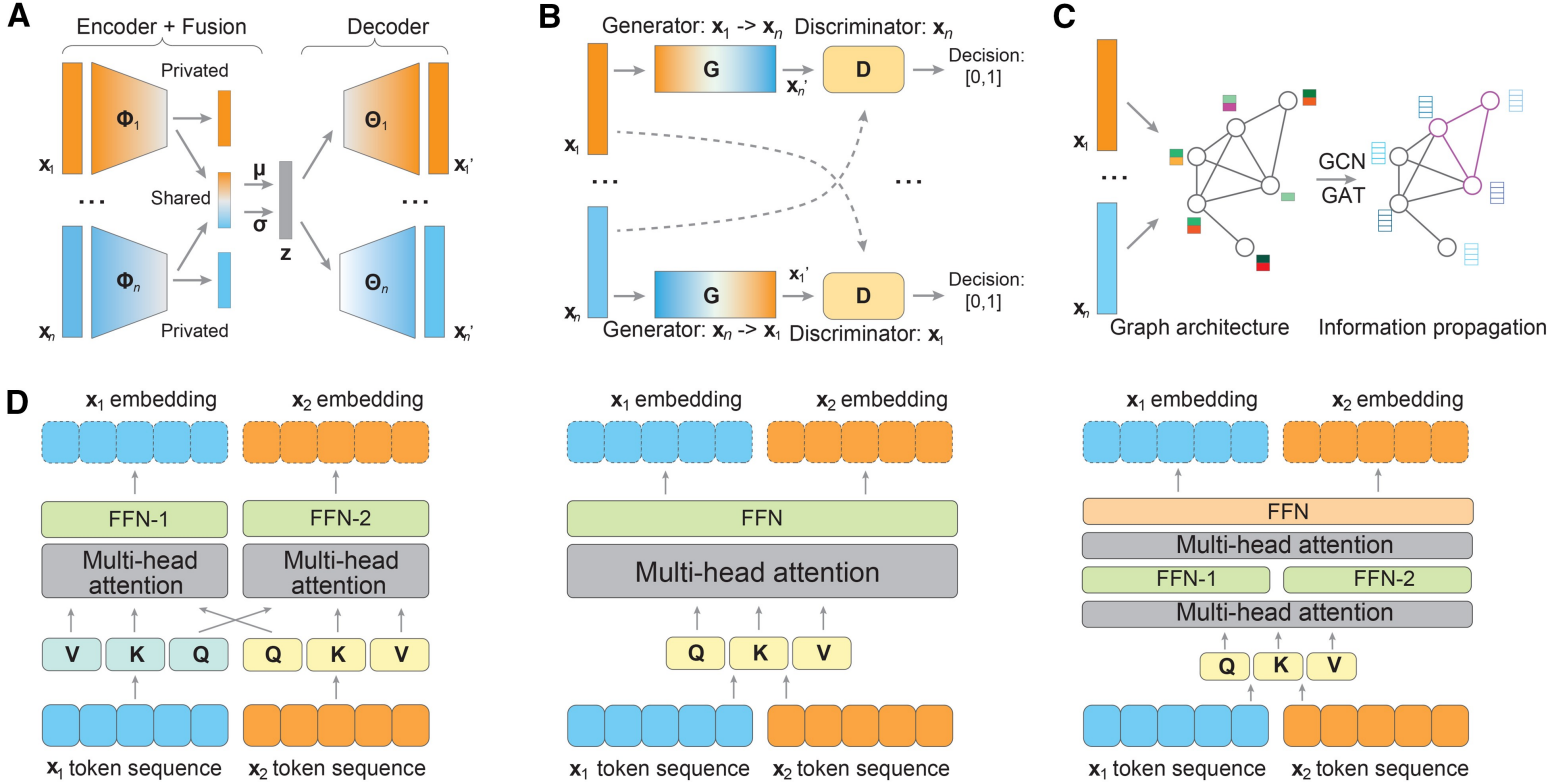
Multimodal representation learning methods **A**.–**C**. Diagram of variational autoencoder (A), generative adversarial network (B), and graph neural network (C) based multimodal representation learning models. **D**. Diagrams of transformer-based multimodal learning models with different attention strategies, cross attention (left), concatenate attention (middle), and modal-specific attention (right). GCN, graph convolutional network; GAT, graph attention network; FFN, feedforward neural network.

Given the heterogeneous nature of multimodal data, performing alignment and integration of these data is extremely challenging. Many methods have been developed to elegantly integrate highly heterogeneous multimodal data to maximize the usage of the information from different modalities to form complementary views (**Figure 2**; **Box 1**).

Box 1 Glossary for computational terms

**CNN:** A type of deep learning model that is particularly effective for processing visual data. It uses convolutional layers to automatically and adaptively learn spatial hierarchies of features from input data.

**RNN:** A type of neural network designed for processing sequences of data, such as time series or text. RNNs can use their internal state (memory) to process sequences of inputs.

**NLP:** A field of artificial intelligence focused on the interaction between computers and human (natural) languages, including tasks such as language translation, sentiment analysis, and speech recognition.

**NER:** A subtask of information extraction that seeks to locate and classify named entities in text into predefined categories such as person names, organizations, and locations.

**VAE:** A type of generative model that learns a probabilistic latent space representation of the input data, allowing for the generation of new data points that are similar to the training data.

**GAN:** A framework for training generative models using a two-player game between a generator and a discriminator. The generator creates new data points, while the discriminator evaluates their authenticity, pushing the generator to improve its outputs.

**Transformer:** A type of deep learning model primarily used in NLP tasks, but also applicable to other sequence modeling problems. Transformers rely on self-attention mechanisms to process input sequences and can capture dependencies between distant positions in the sequence.

**Meta-learning:** The process of improving learning algorithms themselves, rather than improving the performance of a specific model on a specific task.

**Foundation model:** A large-scale pretrained model that can be adapted (fine-tuned) for a wide range of downstream tasks.

**LLM:** A type of foundation model specifically designed for NLP tasks, characterized by its ability to generate human-like text and understand complex language structures.

**GNN:** A type of neural network designed for processing data structured as graphs or networks. GNNs can capture complex relationships and dependencies between nodes in a graph.

**CV:** A field of artificial intelligence focused on enabling machines to interpret and understand visual data, including tasks such as image recognition, object detection, and image segmentation.

**SHAP:** A game theory-based approach to explain the predictions of machine learning models. SHAP values provide a unified measure of feature importance and can be used to explain individual predictions or the overall behavior of a model.

**ViT**: A model that adapts the Transformer architecture to the task of image classification. ViT treats images as sequences of patches and processes them using self-attention mechanisms, achieving state-of-the-art results in various image recognition tasks.

**Zero-shot learning:** The ability of a model to recognize or predict categories that it has never seen during the training phase.

**Few-shot learning:** The ability of a model to perform classification or regression tasks using only a limited number of labeled samples.

*Note*: The authors used ChatGPT to assist in interpreting professional terms in Box 1, then reviewed, edited, and took full responsibility for the content.

### Shallow learning methods

In the early stages of multimodal representation learning, numerous shallow learning methods were developed and utilized. Notable approaches include joint non-negative matrix factorization (jNMF) methods [42], partial least square (PLS) [43], canonical correlation analysis (CCA) [44], and multiple kernel learning (MKL) [45]. The jNMF, PLS, and CCA methods focus on identifying a shared latent space across different modalities through various matrix computation techniques, while MKL seeks to integrate distinct latent spaces from different modalities. Specifically, jNMF applies non-negative matrix factorization on each modality, decomposing them into common and individual factors. PLS maximizes the covariance between decomposed matrices from different modalities to identify separate projections. CCA maximizes correlations between matrices to establish a shared latent space. These methods (jNMF, PLS, and CCA) are frequently employed in multi-omics integration tasks, aiding in the analysis of gene modules and exploring underlying biological mechanisms in the multi-omics latent space [42,46]. In contrast, MKL, a supervised machine learning approach, is commonly used in disease diagnosis and classification tasks within multi-omics studies [47,48].

### Variational autoencoder and generative adversarial network

The variational autoencoder (VAE) is a powerful generative neural network that learns latent representations using a probabilistic approach. It is capable of discovering the underlying structure of the data distribution and facilitating the reconstruction of datasets [49]. This makes it well-suited for inferring joint representations of data from individual biomedical modalities (Figure 2A). In multimodal data fusion, modality-specific or shared encoders were employed to acquire latent embeddings of multimodal data. Subsequently, the data fusion module was used to learn the cross-modality information, while modality-specific decoders were applied to ensure the model’s efficiency in information reconstruction. Based on the position of the layer for the multimodal interaction, the data fusion architectures can be classified into early, intermediate, and later fusion [50]. For functional adaption of VAE into multimodal usage, the mixture of experts (MoE) [51], the product of experts (PoE) [52], and the fusion of MoE and PoE (MoPoE) [53] models were integrated to better infer the joint variational posteriors of the VAE model. Additionally, the development of incomplete modal learning enables more flexible and efficient representation learning in multimodal datasets [54,55]. In addition to the shared representations, the combinations of unimodal-specific representations also exhibited an increment of multimodal representation learning [56]. Moreover, the development of disentanglement learning enables a more interpretable and controllable understanding and generating of biomedical datasets [57].

The generative adversarial network (GAN) is another deep generative model for learning the latent representation and producing artificial datasets of the original datasets [58]. The diagram of GAN consists of two networks: the generator learns to generate increasingly realistic data, while the discriminator network learns to accurately distinguish between real and synthetic data (Figure 2B). The iterations of this adversarial process result in a precise latent embedding of original datasets and high-quality synthetic datasets. In multimodal data learning, cross-or-shared generators were employed to deduce the latent embedding of multimodal data, and discriminators were utilized to discern the authenticity of each generated modality. For instance, CycleGAN applied paired GANs to learn cross-image domains and facilitate translations between different modalities [59]. Huang et al. introduced PoE-GAN, which used a multimodal generator based on PoE to fuse multimodal or unimodal inputs for converting to an image domain [60]. Zhan et al. proposed MGM-GAN, which leveraged a gate emergence mechanism to learn important weights across different modalities, enabling the synthesis of incomplete modalities in MRI [61]. Additionally, Ma et al. introduced the GAN-MVAE model, which integrated GAN and VAE to align the semantic spaces of multiple modalities, enabling zero-shot learning [62]. The capability of VAE and GAN models in multimodal representation learning and their adaptable model frameworks facilitate extensive applications in multimodal biomedical domains. The latent embeddings extracted from complex multimodal data can be employed for various downstream tasks across diverse biomedical applications.

### Graph neural network

Graph neural networks (GNNs) are a type of deep learning model that are specifically designed and well-suited for analyzing complex relationships between objects represented in a graph structure [63] (Figure 2C). In the context of biomedical applications, these graph structures can represent various associations, including gene functions, drug responses, medical image patches, and cellular similarity [64]. The introduction of graph convolutional network (GCN) has enabled GNNs to learn the latent representations of target datasets with a convolution procedure [65]. Additionally, the graph attention network (GAT) structure utilizes attention mechanisms to assess the importance of different edges in the graph while propagating information between nodes [66].

For adapting GNNs into a multimodal usage [67], data points from different modalities are all well-organized in defined graphs, and in contrast to single-modal fusion architecture, GNNs can directly learn the interactions of intra- and inter-modalities simultaneously. The “all-in-one” multimodal graph learning (MGL) architecture [67] introduced a unified framework that included entity identification, topology construction, information propagation, and representation mixing for joint learning of modalities such as images, language sequences, or biological systems. Expanding upon this model by incorporating intricate graph structures and integrating prior knowledge and distributions can lead to more comprehensive representations of the data. Zheng et al. introduced the MMGL framework that applied modal-aware representation learning to extract the intra- and inter-modality representations and then used adaptive graph learning (AGL) to identify associations between patients in disease prediction [68]. Furthermore, integrating biomedical knowledge graphs (KGs) with GNNs can enhance the performance and interpretation of KG inference problems, which will benefit in generating new hypotheses and new drug development [69]. The advantages of GNNs in comprehending multimodal data structures and incorporating existing knowledge contribute to a more robust approach to multimodal representation learning within the biomedical domain.

### Transformer

The Transformer is a neural network architecture that revolutionizes NLP by utilizing an attention mechanism [70], setting it apart from traditional models like convolutional neural networks (CNNs) and recurrent neural networks (RNNs). Unlike these models, Transformers can focus on various parts of an input sequence simultaneously and process data in parallel, which supports long-term memory and comprehensive data representation. This parallelism enhances their capacity for unsupervised data pretraining, enabling effective transfer learning across diverse domains. The success of the Transformer model in NLP inspires the development of LLMs such as BERT [71], GPT-3 [72], Llama 2 [73], Gemini [74], and others [75]. It makes it well-suited to learn the sequence data of biology [76], including the DNA [6], RNA [77], and protein [7] sequence learning, genomic regulatory prediction [78], protein function annotation [79], protein design [80], and further adaptation into medical image [81] and gene expression analysis [13].

Transformers’ unified data input format also enables them to handle multiple modalities flexibly [82], making them highly effective for multimodal learning, especially in visual and language-related tasks [83]. In multimodal transformer models, the data fusion or modality interaction structure can be flexible and different, depending on the timing of interaction, data stream, and attention learning of different modalities [82] (Figure 2D). In ViLBERT, a “co-attention” transformer layer was used to learn the joint representation of the image and natural language and enabled the pretraining and transfer of cross-modality interactions [84]. ViLT introduced a powerful architecture to process both visual and language information with a single transformer; it applied a simple linear projection of image patches to replace the intensive image embedder and confirmed a unified and adaptable Transformer model for arbitrary modalities [85]. In MulT, multiple pairwise cross-modal transformers were merged for learning attention across modalities and then fused embedding from different modalities [86]. With their adaptable architectures and pretraining frameworks, Transformers show promise for advancing multimodal biomedical data analysis, including cross-domain, few- or zero-shot learning with limited clinical datasets in biomedical research [87].

## Applications of AI in multimodal biomedical data analysis

Within the framework of common multimodal representation learning methods, numerous specialized techniques have been developed to apply these approaches to multimodal biomedical data. These methods enhance biomedical image analysis, multi-omics analysis, single-cell analysis, and imaging genomics, offering systematic and comprehensive insights into disease biology and medicine.

### Clinical multimodal data integration

The success of deep learning in computer vision (CV) analysis has significantly advanced its clinical applications in comprehending biomedical images, including CT scans [88], positron emission tomography (PET)-CT scans [89], whole-slide images (WSIs) [90], and MRI scans [91]. This advancement has found extensive application in the fields of brain, cardiac, eye, and cancer diseases [4]. To better understand the pathology of diseases, the concept of multimodal medical image fusion (MMIF) analysis has been proposed, which involves integrating images from different examination methods in the frequency domain, at spatial pixel level, or by merging decisions for individual image modalities [92] (**Table 2**).

**Table 2:** Summary of the applications of multimodal learning in the biomedical field

Method	Field	Year	Data modality	Main focus	Ref.
MCAT	Biomedical image analysis	2021	WSIs, genomics	Integrate WSIs and genomic features for survival outcome prediction	[98]
PORPOISE	Biomedical image analysis	2022	WSIs, genomics	Jointly learn WSIs and molecular features for survival outcome prediction and prognostic feature discovery in pan-caners	[20]
PathIn-NL	Biomedical image analysis	2023	WSIs, genomics	Pathology image classification with pathology and genomics data	[99]
DyAM	Biomedical image analysis	2022	CT scans, PD-1/PD-L1 IHCs, genomics	Integrate radiological, histopathological, and genomic features for immunotherapy response prediction of NSCLC patients	[102]
Custom model	Biomedical image analysis	2022	CT scans, WSIs, genomics	Fuse histopathological, radiologic, and clinic-genomic features to improve risk stratification of HGSOC patients	[103]
Custom model	Multi-omics data analysis	2018	mRNA, miRNA, methylation, clinical information	Integrate multimodal genomic features for liver cancer patients for survival outcome prediction and subtype clustering	[112]
MOGONET	Multi-omics data analysis	2021	mRNA, miRNA, methylation information	Use graph convolutional networks to merge multimodal genomic features for sample classification	[113]
P-NET	Multi-omics data analysis	2021	Mutation, CNV information	Use biologically informed neural network for prostate cancer patient classification and interpretation	[116]
MOVE	Multi-omics data analysis	2023	Genomics, transcriptomics, proteomics, metabolomics, metagenomics, drug usage information	Identify drug–omics associations in type 2 diabetes patients to compare drug similarity and drug effects in multi-omics features	[18]
scMDC	Single-cell analysis	2022	scRNA-seq, ATAC-seq, protein sequencing	Integrating and clustering of single-cell multi-omics datasets and remove batch effects	[119]
GLUE	Single-cell analysis	2022	scRNA-seq, ATAC-seq, methylation sequencing	Integrate unpaired single-cell multi-omics datasets and inferring regulatory interactions	[22]
sciPENN	Single-cell analysis	2022	scRNA-seq, protein sequencing	Integrate CITE-seq and scRNA-seq datasets with protein expression prediction and imputation	[120]
DeepMAPS	Single-cell analysis	2023	scRNA-seq, ATAC-seq, protein sequencing	Use graph transformer framework for learning interactions between cells and genes for cell clustering and gene regulatory network inference	[23]
MIDAS	Single-cell analysis	2024	scRNA-seq, ATAC-seq, protein sequencing	Mosaic integration for single-cell multimodal data	[121]
DestVI	Spatial transcriptomic analysis	2022	Spatial transcriptomics	Multi-resolution deconvolution of single-cell types in spatial transcriptomic data	[126]
STAGATE	Spatial transcriptomic analysis	2022	Spatial transcriptomics	Integrate cellular gene expression and spatial location information	[24]
GraphST	Spatial transcriptomic analysis	2023	Spatial transcriptomics	Use graph self-supervised contrastive learning to integrate spatial and gene expression information	[127]
SpatialGLUE	Spatial multimodal analysis	2024	Spatial transcriptomics, epigenomics, proteomics	Using dual-attention mechanism to integrate spatial information and cross modality information	[130]

*Note*: WSI, whole slide imaging; CT, computed tomography; IHC, immunohistochemistry; CNV, copy number variation; scRNA-seq, single-cell RNA sequencing; ATAC-seq, assay for transposase-accessible chromatin with sequencing; NSCLC, non-small cell lung cancer; HGSOC, high-grade serous ovarian cancer; CITE-seq, cellular indexing of transcriptomes and epitopes by sequencing.

The WSI is a super-high resolution digital image of a histological specimen, which contains gigapixels with detailed cellular morphology information and is commonly utilized in clinical diagnosis [93]. The development of deep learning methods has expanded the applications of digital pathology in education, clinical diagnosis, image analysis, and integration with other clinical diagnostic methods [94]. To perform feature extraction using deep learning on large WSIs, a specific preprocessing procedure was needed. A common approach involved dividing the whole image into smaller patches with a default size, extracting patch-level features, and then aggregating them into slide-level representations. Despite the traditional CNN method [95], the development of the vision transformer (ViT) [96] enabled a self-attention manner to aggregate the patch embeddings and position connectivity in the WSI [97], as well as the hybrid model “Graph-Transformer”. The pathological information extracted using these methods was further utilized for downstream tasks, like sample classification, prognosis prediction, and others. Besides, the multimodal learning of WSIs and genomic datasets is a challenge and opportunity for benefiting the clinical diagnosis and treatment stratification in clinical applications [19]. Chen et al. introduced a multimodal co-attention transformer (MCAT) framework that utilized a genomic-guided co-attention (GCA) layer to learn the attention between WSI instances and genomic pathway embeddings [98], which aided in prognosis prediction and cross-modality interpretations with inferred attention scores. In the PORPOISE framework, Chen et al. introduced a method to integrate WSIs with genomic, and molecular profiles for prognosis prediction and helped identify poor prognosis-related joint biomarkers [20]. Qiu et al. introduced a weakly supervised model PathIn-NL, which included an attention-based hierarchical multimodal fusion module named AHM-Fusion, providing an effective approach to better represent the WSI features and perform information fusions in multimodal learning [99]. The attention-based methods demonstrate their effectiveness in conducting histopathology image-based multimodal analysis.

CT scans or MRI scans are commonly used non-invasive imaging techniques in clinical settings, which provide the structure information of the human body and are intensively used for the diagnosis of cancers, heart disease, and brain injuries. Deep learning methods have been developed for automatically extracting the morphological features of these images and for downstream tasks of lesion detection and segmentation, image enhancement and reconstruction, sample classification, and prognosis prediction [100]. The integration of radiomics and genomics has extended the clinical applications of these images, allowing for the prediction of molecular mutation status from images [101] and the combination of radiomics and genomics features for complementary learning of clinical samples. Vanguri et al. introduced the DyAM framework, which integrates CT images, PD-L1 immunohistochemistry (IHC), and genomic features for predicting clinical responses in non-small cell lung cancer (NSCLC) patients treated with cancer immunotherapy [102]. Boehm et al. comprehensively characterized the multimodal datasets of high-grade serous ovarian cancer and proposed that a combination of histopathological, radiologic, and genomic features can better predict the prognosis of patients and aid in risk stratification [103]. Applying deep learning methods to integrate high-dimensional multimodal data would further improve the current models and would be promising for future clinical applications.

Several other clinical modalities, including EEG, EHRs, and ECG, play essential roles in clinical practice. EHR data have been combined with multi-omics and imaging data to enhance diagnosis and prognosis for a range of diseases, such as Alzheimer’s disease (AD) [104]. Transformer-based frameworks have been employed to combine EHRs with imaging data through attention mechanisms [105]. Additionally, aligning and integrating EEG with functional magnetic resonance imaging (fMRI) data has shown potential for providing deeper insights into human brain dynamics [106]. Integrating ECG with other bioelectrical data, including phonocardiogram (PCG) data, has enabled improved performance of cardiovascular disease diagnosis and would be promising for other biomedical applications [107,108]. Overall, multimodal deep learning represents a significant advancement in precision medicine, promising to further optimize clinical decision-making and patient outcomes.

### Multi-omics data analysis

The central dogma of molecular biology outlines the process of genetic information being transferred from DNA to mRNA to protein, representing a fundamental mechanism for biological information processing [109]. The complex interactions across these omics, as well as metabolomics, lipidomics, glycomics, and others, determine molecular and cellular phenotypes and play a role in the development of human diseases. With the rapid advances in high-throughput technologies and public omics dataset sources, learning the complementary information of samples from multiple omics and identifying disease-related biomarkers and regulatory mechanisms are crucial to the field [110] (Table 2).

Deep learning has been utilized for integrating multi-omics datasets and performing different downstream tasks [111]. In liver cancer research, Chaudhary et al. employed multi-omics data integration to predict the prognosis of hepatocellular carcinoma (HCC) and identify subgroups with significant survival differences [112]. Wang et al. proposed a novel GNN-based method named multi-omics graph convolutional networks (MOGONET) for biomedical sample classification and identified the subgroup-specific biomarkers [113]. Apart from data fusion with deep learning, understanding the underlying regulatory mechanism and identifying target drugs are crucial for utilizing multi-omics datasets [114]. XOmiVAE introduced an interpretable deep learning model to integrate high-dimensional omics data and explain the contributions of genes in a supervised and unsupervised manner [115]. P-NET applied the hierarchical pathway information to construct a sparse deep neural network for inferring the disease status-specific molecular alterations, which aids in disease diagnosis and drug design for prostate cancer [116]. We also developed the TMO-Net model for incomplete multi-omics data learning of cancer data and adapted it into multiple downstream tasks [55]. Additionally, Froguel et al. introduced a multi-omics variational autoencoders (MOVE) framework for identifying the associations between drug usage and multi-omics data features, helping characterize the drug effects on type 2 diabetes [18]. These methods demonstrate the potential of multi-omics integration in personalized medicine, particularly in cancer and metabolic diseases, by enhancing diagnostic and therapeutic capabilities. Meanwhile, interpretable models enhance our ability to discern the significance of multi-omics features and connect them to biological functions or clinical outcomes. This interpretative layer is essential for validating findings, identifying disease mechanisms, and translating discoveries into actionable insights in fields such as oncology and pharmacology, ultimately supporting personalized treatment and biomarker discovery.

### Single-cell data analysis

With the advancement of high-throughput single-cell capture and sequencing techniques, multiple types of single-cell omics data have been generated, including transcriptome, chromatin, DNA methylation, histone modification, and others [21]. Integrating these datasets and establishing interactions between them at different levels are crucial for the successful application of these sophisticated methods [117]. A NeurIPS competition has found three key tasks, including predicting one modality from another, matching cells between modalities, and jointly learning representations of cellular identity, aiming for adapting deep learning to advance single-cell analysis and expand the understanding of cellular biology [118] (Table 2).

For integrating multi-omics datasets from paired or unpaired single cells, Lin et al. introduced the scMDC method, which utilized an end-to-end autoencoder model to learn the joint embeddings of paired single-cell multi-omics datasets [119]. Cao et al. introduced the graph-linked unified embedding (GLUE) framework for integrating unpaired single-cell multi-omics datasets guided by a linked graph representation of omics features and inferred the regulatory interactions across modalities [22]. Lakkis et al. presented the sciPENN framework, which integrated and imputed incomplete protein expression from multiple CITE-seq datasets and further integrated other scRNA-seq datasets and transferred cellular labels across modalities [120]. He et al. introduced MIDAS, a deep-learning method that enabled mosaic data integration of single-cell multimodal data and knowledge transfer into new datasets [121]. DeepMAPS constructed a mixed graph representation of cell–gene networks, applied the heterogeneous graph transformer (HGT) model to capture the importance between cells and genes, and further inferred the gene regulatory networks to specific cell types [23]. These studies suggest the efficacy of graph-based representation methods in capturing regulatory interactions across single-cell multi-omics data.

The rapid development of spatial transcriptomics has provided information on local tissue contexts and adjacent cellular interactions [122,123]. Furthermore, the combination of CITE-seq with spatial sequencing has extended the multi-omics utility in the local context [124]. Deep learning methods have been applied to extract the interactions between cellular location and molecular phenotypes [125]. DestVI proposed a framework that jointly learned the latent representations of scRNA-seq datasets and spatial spot expression datasets to deconvolute cell-type proportions and related transcriptional states in single spots [126]. STAGATE utilized the graph attention autoencoder model to learn the integrated spatial and gene expression profiles [24]. Long et al. introduced the GraphST framework, which combined GNN and contrastive learning to integrate information within spatial transcriptomic datasets, across tissue sections and scRNA-seq datasets [127]. Zhou et al. [128] developed the graph attention neural network STAligner to integrate spatial transcriptomic data and scRNA-seq data and map scRNA-seq data to specific spatial locations based on the location information provided by spatial transcriptomic data. Tangram [129] also integrated multimodal data for mapping, and it can also use supervised learning methods to infer the distribution of cell types in spatial locations from known cell types or gene expression signatures. Additionally, SpatialGLUE applied a GNN to integrate spatial multi-omics data [130], which captured more anatomical details including undiscovered cell types, and provided more accurate information on spatial domains. The development of single-cell multi-omics methods and the growth of single-cell datasets created a well-suited scenario for applying deep learning methods in single-cell analysis, which could further lead to advancements in the field of drug discovery, therapeutic targets, and digital health [2,131].

### Genotype–phenotype association analysis

Identifying the associations across different data domains is a major challenge in multimodal data integration, such as identifying associations between imaging and genomic data, referred to as radiogenomics. Traditional approaches fall short in extracting these associations, limiting the potential applications of imaging genomics in precision medicine. In contrast, AI methods show promise in imaging genomics by learning complex cross-modal relationships. While some deep learning approaches have focused on detecting molecular alterations in image data [132], most existing studies have centered on inferring spatial transcriptome or bulk transcriptome information from histopathology images [133–136]. Although these approaches demonstrated potential in using cost-effective imaging data as surrogates for molecular information in clinical settings, they fall short in comprehensively capturing molecular omics data and uncovering the underlying biological mechanisms linking imaging and omics data. Advanced multimodal learning methods offer significant promise for revealing the biological code underlying complex image phenotypes and molecular omics data, advancing disease understanding.

Similarly, identifying associations between omics data and clinical information is essential for discovering disease biomarkers and identifying risk factors. However, linking clinical phenotypes to omics data, especially single-cell and spatial omics data, is challenging. Since these data are often high-dimensional and cell-level, traditional methods have struggled to connect them with individual-level clinical phenotypes, such as disease classification and status. To address this, an attention-based neural network, ScRAT, was developed to bridge scRNA-seq data with clinical phenotypes [137], facilitating phenotype-specific cell-type identification and high-resolution disease classification. For spatial omics analysis, a graph-based deep learning algorithm has been developed to predict patient outcomes from spatial proteomics profiling [138], facilitating the identification of disease–phenotype-specific tumor microenvironment patterns. Future research that incorporates more phenotype information with multi-omics data holds promise for advancing our understanding of disease mechanisms and enabling precise disease subtyping.

## Challenges in AI-based biomedical multimodal data analysis

Despite existing advancements, there are challenges when performing AI-based biomedical multimodal data analysis. Tackling these challenges would facilitate extensive biomedical discoveries.

### Data challenges

**Figure 3:**
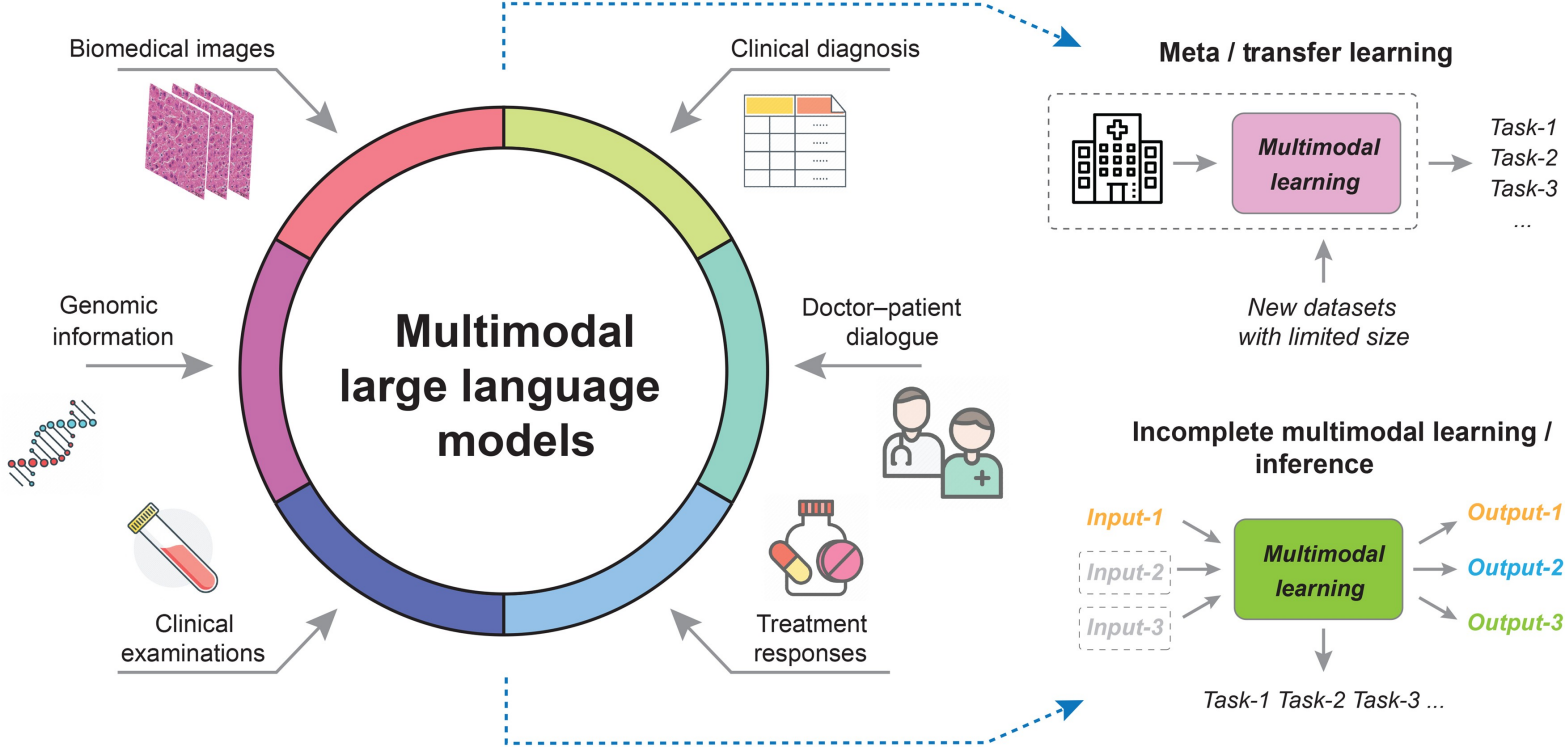
Diagram of the future directions and applications in biomedical multimodal learning The incorporation of multimodal data, including biomedical images, genomic information, clinical diagnosis, doctor–patient dialogue, treatment responses, and clinical examinations, into multimodal large language models would be future directions for biomedical multimodal learning. Challenges including limited data availability and incomplete multimodal data will be resolved by meta/transfer learning and incomplete modal learning/inference.

Multimodal learning has been widely and successfully applied in natural language and vision domains, but its application in multimodal biomedical datasets presents significant challenges due to the diversity of data sources, ranging from molecular profiles to image examinations of human bodies, and may have a high rate of missing information in each modality [1] (**Figure 3**). Additionally, in specific modalities, the data acquisition and preprocessing procedures can be different, which hampers the standardization and interoperability of data across studies. Multiple dataset portals have been established to maintain the multimodal biomedical data, including The Cancer Genome Atlas (TCGA) [139], International Cancer Genome Consortium (ICGC) [140], Molecular Taxonomy of Breast Cancer International Consortium (METABRIC) [141], and The cBioPortal for Cancer Genomics (cBioPortal) [142] for cancer research, the Diabetes Remission Clinical Trial (DiRECT) [143] consortium for type 2 diabetes, the Alzheimer’s Disease Neuroimaging Initiative (ADNI) [144] dataset for AD, and other portals like UK Biobank [145] for general digital health records, but the data size of specific cancer types or diseases remains limited, which may impede neural network training.

Multiple approaches have been developed to address the challenges of data scarcity and data missing. Self-supervised learning is an important approach for addressing the challenge by fully leveraging the unlabeled data. Based on contrastive learning and data augmentation, the self-supervised learning approach can learn robust data embeddings from unlabeled data and the trained model can be fitted to many downstream tasks [146,147]. Typical examples include Transpath [148] and CS-CO [149] for histopathological image analysis, which reduced the costs for histopathological image annotations. Besides, deep learning models have been designed to enable applications in incomplete modality learning. The MeLIM method proposed a framework to impute the missing modalities via a GAN framework and learned the joint sample representations [150]. The M^3^Care method learned the unimodal representations, constructed the similarity graph of patients, and then imputed the latent space of missing modality in different patients [151]. Tu et al. introduced the cross-linked unified embedding (CLUE) model for utilizing the cross-encoders between modalities to learn a comprehensive representation of incomplete datasets [152]. Hou et al. applied a novel hybrid graph convolutional network (HNCG) model and online masked autoencoder to learn the intra- and inter-modal interactions across multimodal biomedical datasets and addressed missing modality learning [153]. Furthermore, cross-modal data synthesis provides another potential solution. Caroline et al. developed a cross-modal analysis method that enables the imputation of hard-to-acquire cardiac MRIs from easy-to-acquire ECG [154]. Wang et al. developed a joint learning framework providing cross-modal synthesis between MRI and PET data and improving the diagnosis of AD [155]. GAN-based networks were applied in cross-modality synthesis from CT to PET image, which achieved an improvement in automated lesion detection [156]. Carrillo-Perez et al. developed a cascaded-diffusion model to synthesize WSIs from RNA-seq data, which accurately preserved the distribution of cell types in WSIs [157]. However, the applications of these synthetic data in model training require further research.

Privacy protection is another significant challenge in the deep learning of biomedical datasets [25], which requires the development and deployment of privacy-preserving deep learning methods. One approach is federated machine learning [158], which allows the training of individual datasets locally and updating the core main model without accessing the private data. Secure multiparty computation (SMPC) is another approach for privacy-preserving with a cryptographic framework [159]. For instance, Hie et al. proposed a framework for allowing predicting drug–target interactions based on private datasets from individual entities [160]. Additionally, the synthetic data generators were applied for building similar replicas of the original private datasets, while maintaining consistent statistical properties [161,162].

### Biomedical data interpretation

Machine learning models have proven to be successful in various biomedical learning tasks. Despite their complex model design and superior task performance, understanding and uncovering the underlying decision-making (interpretation) process is crucial in biomedical learning (Figure 3), particularly in mechanism identification, drug design, and treatment selection [27]. In general, there are two main streams in biological interpretation: one is biology-informed neural network design, and the other is *post-hoc* model learning. The common approach for biological model design is to construct a neural network architecture that is restrained by biological pathways, specifically connecting target gene nodes with pathway nodes, including models of P-NET [116], pmVAE [163], VEGA [164], and LDVAE [165]. For instance, Lotfollahi et al. introduced expiMap, which used biological domain databases to learn cellular gene programs and was designed for inferring *de novo* gene programs, which enables more efficient cellular annotation and querying of new single-cell datasets [166]. The major challenge in biology-informed neural network design is the bias of biology design, which restricts knowledge extraction and is unavailable for multimodal usage due to the limited number of curated biology networks in specific modalities. Moreover, disentanglement learning is applied in biomedical learning for identifying disease-related latent variables and generating synthetic datasets to aid in mechanism validation. Yu et al. proposed MichiGAN, which combined VAEs and GANs to learn the disentangled representations of single-cell datasets and generate single-cell datasets with biological insights [167]. By defining a set of latent-related data regulation roles, experts can then uncover the underlying causalities and help the biomedical understanding of the datasets.

Gradient- and perturbation-based methods have been extensively used for *post-hoc* interpretation of deep learning models [168]. In gradient-based methods, the contributions of input features in individual modalities were estimated with a significant score. Jha et al. proposed the enhanced integrated gradients (EIG) method [169], which identified significant splicing code features in the liver and was adapted for identifying common transcriptional signatures of cancers [170]. For perturbation-based methods, XOmiVAE calculated the Shapley Additive exPlanations (SHAP) value [171] of input gene features and then identified the most important genes for the sample classification [115]. The further usage of this method in unsupervised sample clustering, allows for an activated-based interpretation of novel clusters. The MOVE framework utilized a perturbation-based approach to identify significant drug-related multimodal features in type 2 diabetes, providing a framework for interpreting biomedical multimodal datasets [18]. Furthermore, understanding cross-modality interactions is a critical challenge in multimodal learning, such as the downstream effects of gene mutations in transcriptomic or proteomic regulations. Liang et al. introduced the MULTIVIZ framework for understanding the interactions between image and word embedding in multimodal learning [172], but associated applications in multimodal biomedical data learning are still limited. Besides, more efforts are required to validate the results of *in silico* interpretation learning. Chen et al. proposed Explanation Verification, which utilized synthetic datasets to confirm the intended logic of uncovered biological mechanisms for knowledge discovery in computational biology learning [29].

### Cross-scale data integration

Biomedical data often span various scales, such as clinical data, bulk omics data, and single-cell omics data. Integrating these cross-scale datasets is crucial yet challenging for generating meaningful biomedical insights. To integrate bulk and single-cell transcriptome data, researchers have employed the β-VAE method to deconvolute bulk data into cell-type-specific expression profiles, addressing the ‘omission’ issue in single-cell sequencing [173]. In other cases, such as integrating multi-scale histopathology images, methods like cross-scale attention mechanisms and multiple instance learning (MIL) have been effective [174]. The MIL approach also shows potential in integrating other types of cross-scale data [175]. For cross-scale biomedical imaging, integration can often be achieved using shallow learning methods, though challenges remain in accurately registering and aligning cross-scale images [176]. RAPHIA, an end-to-end algorithm based on a geometry-consistent generative adversarial network (GcGAN), offers a promising solution for the registration of MRI and histopathology images, enabling cross-scale analysis of medical and histopathology images [177]. Further research is needed to enhance cross-scale information integration and alignment, which would deepen our understanding of biological systems.

## Future directions of AI and biomedical multimodal data analysis

Given the challenges in analyzing biomedical multimodal data, we outline key future directions for AI in this field (Figure 3).

### Meta and transfer learning

While the success of deep learning in digital health has inspired the development of models for various diseases and tasks, the limited size of cohorts for certain diseases poses a significant challenge for efficient training and validation of these models. To overcome this problem, few- or zero-shot learning approaches, such as meta-learning and transfer learning, have been proposed to enable adaption in small-size datasets [178]. The pretrain-and-finetune framework has shown its effectiveness in computer vision learning, neural language processing, and biomedical learning for prognosis prediction [179] and cancer dependency prediction [180]. In single-cell analysis, Lin et al. proposed the scJoint framework [181], which applied semi-supervised learning of scRNA-seq datasets with cell-type information, and further transferred cellular annotation to scATAC-seq datasets and multimodal data integration. Lotfollahi et al. applied transfer learning for mapping query single cells with a decentralized and iterative updating reference model [12].

Meta-learning, a technique where models learn to adapt to new tasks efficiently, also shows promise in biomedical applications. For instance, Qiu et al. introduced a meta-learning framework to identify the sample representation by integrating the trained models across multiple tasks and performing prognosis prediction with limited training data [182]. Cho et al. further adapted a similar method to the multimodal survival analysis [183]. Furthermore, meta-learning has been applied to learn the interactions between cell-line phenotypes with the drug responses, and further transferred them into clinical context, enabling prediction on a limited number of human tumor samples [184]. The general landscape of biomedical multimodal datasets, including data scarcity, large unlabeled data, and missing modalities, necessitates more flexible deep learning methods for extracting and transferring knowledge from unlabeled data. The promise of meta and transfer learning in the biomedical learning field offers an avenue for solving these issues and expanding the applicability of deep learning to various biomedical tasks.

### Foundation models

The success of LLMs in the field of neural language processing, as well as their remarkable ability to solve complex tasks, has inspired researchers’ enthusiasm for adapting these models in the biomedical field [185]. This includes constructing large foundation models for genomics [186], clinical images [187], and clinical text learning [188]. Moor et al. introduced a paradigm for foundation model application in medical AI, named generalist medical AI (GMAI) [16]. They proposed that the GMAI model should be capable of adapting simply to new tasks, flexibly combining input and output biomedical modalities, incorporating medical knowledge, and enabling result reasoning. For clinical usage, this model should be able to automatically generate disease reports, summarize patient-clinician conversations, suggest medical interventions, and others. In biological applications, this model would fuse multimodal datasets such as genomics, epigenomics, proteomics, and clinical information, and integrate with biologically informed databases to reveal the molecular regulations of specific clinical phenotypes, which can aid in clinical diagnosis, drug response prediction, and drug design [16].

The application of foundation models in single-cell analysis has greatly advanced the understanding of complex biological systems. scGPT [14] focuses on processing these high-dimensional and sparse RNA data to capture complex relationships between cells, thereby supporting transfer learning across datasets and even across species. GeneFormer [189] focuses on the inference of gene function and regulatory networks, which has certain advantages in exploring the role of specific gene sets and gene clusters in cells and paves the foundation for omics-guided personalized therapy, but its generalization capability needs to be improved. scFoundation [15] strengthens the modular design, aiming to combine different modules to adapt to a variety of single-cell tasks, and is very flexible and scalable. Yang et al. developed GeneCompass, a knowledge-informed cross-species foundation model for understanding the universal gene regulatory mechanisms [190]. However, large-scale single-cell data models still need to face problems such as effective integration of massive multimodal data and model interpretability. In addition, data on rare cell types are scarce and are overlooked by models because they are submerged in mainstream cells.

Emerging foundation models are capable of manipulating large-scale image and text data. Several foundation models have been developed for clinical image data and clinical reports. UNI [191], Prov-GigaPath [192], and Virchow [193] were pretrained solely on pathology image data to perform cancer classification, diagnosis, and prognosis. PLIP [194] and CONCH [195] were pretrained on paired pathology images and text descriptions using contrastive learning methods. CHIEF considered the tissue origin as a label by applying a weakly supervised method during pretraining [196]. BiomedGPT integrated different modalities of clinical images and corresponding clinical reports using unified tokenization methods [197]. Some other multimodal foundation models were developed to provide multiple disease screening and detection. For example, EyeCLIP was pretrained on multimodal ophthalmic images for the detection of multiple diseases [198]. The advancements of multimodal foundation models have improved current disease diagnosis and prognosis prediction. However, no foundation models to date have incorporated image data with omics data, which limits the clinical usage of image information in guiding precision medicine.

The significantly high costs and extensive data collection requirements for LLM training present major challenges in establishing foundation models for biomedical learning, particularly in collecting paired multimodal datasets from individual patients [199]. To address these problems, one approach is to combine prompt tuning to enhance LLMs for learning medical knowledge and adaptation for biomedical applications [200]; another approach is to infuse knowledge graphs into the LLMs [201]. Additionally, the scaling of inference and the knowledge distillation of reasoning-focused LLMs, such as DeepSeek-R1 [202], show promise in advancing clinical applications by providing expert-level recommendations at an affordable cost. Furthermore, another critical feature of LLMs is their capability to use tools for automated online search, task design, and experiment execution [203–205], which exhibits their further capabilities in automatically collecting and preprocessing biomedical datasets, designing bioinformatic analyses to identify disease-related molecular regulations, conducting literature reviews for result interpretation, and proposing potential interventions for disease treatment. Therefore, integrating LLMs with biomedical multimodal learning, including dataset collection, model fusion, and causality inference, holds great promise for accelerating the development of biomedical research.

## Concluding remarks

The growth of biomedical examination methods has significantly expanded our understanding of human diseases, ranging from molecular to body scales, resulting in the generation of thousands of datasets. These datasets have enabled the utilization of AI techniques in various downstream tasks, such as sample classification, prognosis prediction, image lesion detection, image segmentation, and others [206], which have demonstrated higher precision and efficacy in clinical settings [94]. Moreover, the advancement of multimodal measurement methods has enabled the development of algorithms capable of integrating various modal datasets, learning the joint data representations, and identifying associations across cross-modal features. Traditionally, fusion analysis of multimodal data relies on linear correlation approximations, often resulting in an incomplete understanding of the underlying mechanisms. However, the emergence of AI techniques has effectively addressed this limitation. Additionally, deep learning technologies have overcome challenges related to parameter estimation in complex data distributions, a crucial aspect of biomedical data analysis. This progress allows for a more comprehensive understanding of underlying regulatory mechanisms, highlighting the potential of multimodal approaches in biomedical data analysis [1].

Various challenges remain in applying deep learning methods to biomedical multimodal learning. The high costs of biomedical data generation, difficulties in patient follow-up, and privacy restrictions limit the size of training datasets and lead to missing data modalities [28]. These issues pose significant data challenges in biomedical multimodal learning and necessitate the design of models capable of handling incomplete modal learning and applicable to small cohort samples. Interpreting deep learning models is another major challenge in biomedical multimodal learning; understanding the critical roles of multimodal features in the decision-making process of deep learning models can help identify the biological regulatory mechanisms of potential treatment targets, and assist in the decision of medical intervention and drug design [29]. Additionally, integrating biomedical knowledge into deep learning models presents a challenge. While multiple deep learning methods have been developed to integrate biological pathway information with neural network architectures [116,166], these methods are still limited by the predefined pathway annotations. Moreover, fusion strategies used in deep learning methods affect the cross-modality feature interpretation in multimodal learning, which is crucial in understanding the regulatory process of biological systems. Although the perturbation-based method has been effective in characterizing the most drug-responsive omics features [18], there is still a need for further research and discussions to improve cross-modal interpretation by refining model designs.

The success of LLMs has revolutionized AI learning in natural language and image processing [75] and has been extended into the learning of biological sequence datasets [80]. The enormous scale of neural networks and vast training datasets enable these models to quickly adapt to new tasks. Moor et al. proposed a framework of the GMAI, a foundation model designed to handle most clinical tasks, aiming to provide high-quality medical support to both patients and clinicians [16]. Similarly, a foundation model is needed for biomedical multimodal learning that enables multimodal data query and imputation, leverages biomedical knowledge, facilitates joint representation learning, generates hypotheses, and assists with clinical decision-making, drug design, and understanding of human diseases.

More public biomedical datasets and a collaborative community effort will be required to realize such a foundation model. Additionally, the scenario of limited cohort samples for training and application in biomedical multimodal models necessitates the incorporation of few-shot or zero-shot learning techniques in model design. Techniques such as meta-learning and transfer learning can be employed to learn general data representations from other large datasets [178,181]. Despite applying advanced AI techniques in biomedical data analysis, it is important to acknowledge the limitations of AI and deep learning in life sciences and clinical medicine. Current successful applications are primarily focused on solving scientific problems within these fields, with relatively few innovations in deep learning computational methods themselves. Even fewer advancements have emerged in AI algorithms inspired by life sciences. Addressing these gaps represents a critical direction for future research regarding AI applications in multimodal biomedical data analysis.

In summary, this review highlights the role of AI in biomedical multimodal data analysis, covering multimodal biomedical data, multimodal representation learning methods, and their applications for a range of biomedical multimodal data analysis tasks, including data integration, multi-omics analysis, single-cell analysis, and genotype–phenotype association studies. Challenges in AI-based biomedical multimodal data analysis include handling diverse data types, interpreting biomedical data, and achieving effective cross-scale data integration. Future efforts should focus on developing foundational models and employing meta- and transfer-learning techniques for cross-scale multimodal biomedical data analysis. These necessitate collaboration in data collection, model design, and external validation to fully leverage these biomedical multimodal datasets and improve human health outcomes.

## CRediT author statement

**Junwei Liu:** Conceptualization, Investigation, Writing – original draft. **Xiaoping Cen:** Investigation, Writing – original draft, Writing – review & editing. **Chenxin Yi:** Investigation, Writing – original draft, Writing – review & editing. **Feng-ao Wang:** Investigation, Writing – original draft. **Junxiang Ding:** Investigation, Writing – original draft. **Jinyu Cheng:** Investigation, Writing – review & editing. **Qinhua Wu:** Investigation, Writing – original draft. **Baowen Gai:** Investigation, Writing – review & editing. **Yiwen Zhou:** Investigation, Writing – original draft. **Ruikun He:** Investigation. **Feng Gao:** Supervision, Conceptualization. **Yixue Li:** Supervision, Conceptualization. All authors have read and approved the final manuscript.

## Competing interests

Ruikun He, an employee of BYHEALTH Institute of Nutrition & Health, declares that she has no competing financial, personal, or professional interests related to the manuscript. All the other authors have declared no competing interests.
